# Neuromyelitis in a Patient with Rheumatoid Arthritis: A Case Report

**DOI:** 10.34172/aim.28804

**Published:** 2024-05-15

**Authors:** Fuyong Qiang, Zhi Li, Lanfang Chen, Dan Xuan, Jun Sheng

**Affiliations:** ^1^Department of Rheumatism and Immunology, The First Affiliated Hospital of Wannan Medical College, Wuhu, Anhui, China

**Keywords:** Aquaporin-4, Neuromyelitis, Rheumatoid arthritis

## Abstract

A patient with longstanding rheumatoid arthritis (RA) complained of spinal cord symptoms after RA relapse. Contrast MRI demonstrated neuromyelitis in the upper thoracic spinal cord, and anti-aquaporin-4 (anti-AQP4) antibody was positive in the serum and cerebrospinal fluid (CSF). Neuromyelitis optica spectrum disorder (NMOSD) was diagnosed after excluding central nervous system (CNS) infection and tumor, and spinal cord symptoms were relieved after high dose of glucocorticoid and immunosuppressant were initiated for treatment.

## Introduction

 Rheumatoid arthritis (RA) is a systemic autoimmune disease that mainly involves peripheral joints. Nerve damage occurs in about 20% of patients. The peripheral sensory and motor nerves are commonly involved, and the most common symptoms are limb weakness and sensory abnormalities due to vascular ischemia, axonal degeneration, and neuronal demyelination, which are caused by vasculitis.^1^ Central nervous system (CNS) involvements in RA patients includes meningitis, optical atrophy, and cerebral vasculitis.^2,3^ In addition, cervical myelopathy caused by cervical spine cord compression is the most common among these alterations in patients with longstanding RA.^4^ However, neuromyelitis in RA is rare. Here, we present a case of RA with neuromyelitis.

## Case Report

**Figure 1:**
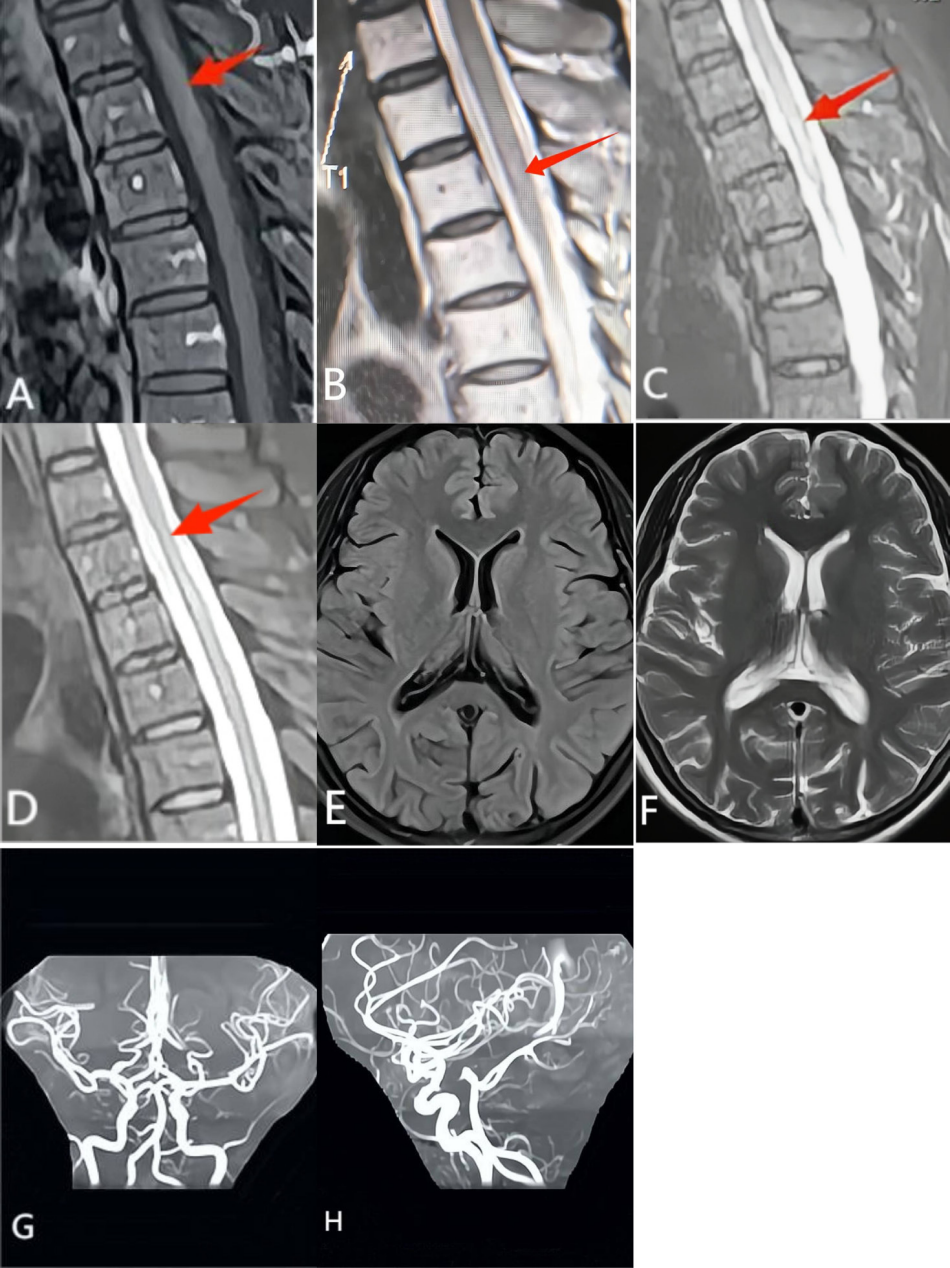
Magnetic Resonance Image (MRI) of the Upper Thoracic Spinal Cord in the Patient. (A-B) MRI shows longitudinally extensive T1-weighted and T2-weighted hyperintensity in the upper thoracic spinal cord (T1-T4) (red arrow). (C) Contrast MRI T2-weighted demonstrates enhanced high signal change (red arrow). (D) High signal change was significantly improved after treatment one year later (red arrow). (E-H) No abnormal changes were observed in either brain MRI or MRA

 A 61-year-old female complained of swelling and pain in the proximal interphalangeal joints and metacarpophalangeal of both hands accompanied by morning stiffness > 1 hour per day for ten years. She was diagnosed as RA as both rheumatoid factor (RF) and anti-cyclic citrullinated protein (anti-CCP) antibody were positive. The patient refused to take methotrexate, and low-dose prednisone combined with leflunomide and sulfasalazine were prescribed for treatment. The joint symptoms were relieved after three years of treatment and the patient stopped taking all drugs spontaneously. Swelling and pain of metacarpophalangeal joints, wrist joints, and ankle joints re-occurred one year later accompanied by acupuncture pain, pruritus and persistent superficial hypoesthesia on the back of bilateral nipple plane. No skin lesions were observed in this area and the patient did not exhibit any systemic signs such as high fever, weight loss, heart failure, or gastrointestinal bleeding. Laboratory investigations revealed erythrocyte sedimentation rate (ESR) of 94 mm/h, C-reactive protein (CRP) of 47.5 mg/L, and IgG of 23.73 g/L. IgM-RF and anti-CCP antibody were at 86.5 IU/mL (normal < 20) and 386.0 U/mL (normal < 25), respectively. Complete blood count and tests for liver function, renal function, tumor markers, urinalysis, cardiac enzymes, complement, PPD-test and T-spot were all normal. Tests for hepatitis, syphilis, HIV, and autoantibody profile were all negative. Schirmer’s test and Saxon test showed scores of 12 mm/5 min and 3.5 g/2 min, respectively. The patient refused to undergo sialography, and salivary gland ultrasonography was performed which indicated mild atrophy of bilateral parotid glands. Lip biopsy revealed no significant lymphocyte infiltration. No abnormal changes were observed in brain MRI or MRA (Figure 1E-H). MRI showed longitudinally extensive T1-weighted and T2-weighted hyperintensity in the upper thoracic spinal cord (T1-T4) and contrast MRI T2-weighted demonstrated enhanced high signal change (Figure 1A-C). Biochemical, routine and microbial tests of cerebrospinal fluid (CSF) were negative. Immunoglobulin levels were normal in the CSF: IgA 4 mg/L (normal range 0-6 mg/L), IgG 25 mg/L (normal range 10-40 mg/L), IgM 7.4 mg/L (normal range 0-13 mg/L). In addition, the CSF was negative for oligoclonal IgG bands. Anti-aquaporin-4 (AQP4) antibody was positive and anti-myelin oligodendrocyte glycoprotein antibody was negative in the serum and CSF. The patient was diagnosed as RA complicated by neuromyelitis optica spectrum disorder (NMOSD); her expanded disability status scale (EDSS)^5^ was 2.0. She was treated with 1000mg intravenous methylprednisolone for 3 days and 500mg intravenous methylprednisolone for 3 days. Maintenance treatment included a tapering high dose of oral prednisolone. In addition, intravenous cyclophosphamide 1g once a month for half a year was initiated then altered to oral azathioprine for maintenance treatment. Acupuncture pain, pruritus, and superficial hypoesthesia were relieved after treatment. Thoracic spinal cord MRI demonstrated that myelitis was significantly improved one year later (Figure 1D).

## Discussion

 In the present case, the patient had typical joint symptoms as well as positive RF and anti-CCP antibody, which conformed to the 2010 diagnostic criteria for RA established by the American Academy of Rheumatology/European Alliance Against Rheumatism (ACR/EULAR).^6^ The joint symptoms were relieved after treatment. However, the patient demonstrated involvement of the spinal cord besides joint symptoms when RA re-occurred. Contrast MRI indicated neuromyelitis in the upper thoracic spinal cord and anti-AQP4 antibody was positive in the serum and CSF. No evidence of infection or tumor was found in the CNS. Therefore, the patient conformed to the 2015 diagnostic criteria for NMOSD.^7^ Systemic lupus erythematosus (SLE) and Sjogren’s syndrome (SS) are the most common reasons in connective tissue disease-associated neuromyelitis as these diseases can lead to spinal cord vasculitis by forming many immune complexes.^8^ However, SLE and SS could be excluded in this case as a result of negative autoantibody profile, normal hemogram, negative urine analysis, negative Schirmer’s test and Saxon test as well as negative lip biopsy. Visual impairment was reported as the prominent manifestation in some previous reports.^9,10^ However, spinal cord involvement was the main manifestation in this case. Anti-AQP4 antibody is closely associated with occurrence of NMOSD,^11^ the proportion of positive anti-AQP4 antibody and the titer of anti-AQP4 antibody were all increased in NMOSD, but this antibody was not detected in SLE and RA patients in a previous study.^12^ Regarding the genetic background, the HLA-DRB1 *04 allele increases susceptibility to seropositive RA and NMOSD,^13,14^ and we speculate that a common genetic locus leads to the simultaneous occurrence of both diseases.

 We reported a rare case of RA complicated with NMOSD. However, we could not conclude whether RA and NMO were two isolated disorders or NMO was caused by the RA relapse. The relationship between RA and NMO needs further studies for clarification.
